# Case report: Oligodendroglioma, IDH-mutant and 1p/19q-codeleted, associated with a germline mutation in *PMS2*

**DOI:** 10.3389/fonc.2022.954879

**Published:** 2022-08-02

**Authors:** Mythili Merchant, Margarita Raygada, Ying Pang, Martha Quezado, Mark Raffeld, Liqiang Xi, Jung Kim, Manoj Tyagi, Zied Abdullaev, Olga Kim, Zach Sergi, Tina Pillai, Byram Ozer, Kareem Zaghloul, John D. Heiss, Terri S. Armstrong, Mark R. Gilbert, Kenneth Aldape, Jing Wu

**Affiliations:** ^1^ Neuro-Oncology Branch, Center for Cancer Research, National Cancer Institute, Bethesda, MD, United States; ^2^ Laboratory of Pathology, Center for Cancer Research, National Cancer Institute, Bethesda, MD, United States; ^3^ Surgical Neurology Branch, National Institute of Neurological Disorders and Stroke, Bethesda, MD, United States

**Keywords:** lynch syndrome, *PMS2*, CNS, oligodendroglioma, IDH-mutant and 1p/19q-codeleted

## Abstract

Most tumors, including brain tumors, are sporadic. However, a small subset of CNS tumors are associated with hereditary cancer conditions like Lynch Syndrome (LS). Here, we present a case of an oligodendroglioma, IDH-mutant and 1p/19q-codeleted, and LS with a germline pathogenic *PMS2* mutation. To our knowledge, this has only been reported in a few cases in the literature. While the family history is less typical of LS, previous studies have indicated the absence of a significant family history in patient cohorts with *PMS2* mutations due to its low penetrance. Notably, only a handful of studies have worked on characterizing *PMS2* mutations in LS, and even fewer have looked at these mutations in the context of brain tumor development. This report aims to add to the limited literature on germline *PMS2* mutations and oligodendrogliomas. It highlights the importance of genetic testing in neuro-oncology.

## Introduction

Oligodendroglioma, IDH-mutant and 1p/19q-codeleted, is a subset of diffuse gliomas that primarily develop sporadically. Very few patients with oligodendroglioma have been associated with a hereditary cancer predisposition syndrome. According to the most recent CBTRUS Statistical Report, these tumors have an adjusted annual incidence rate estimated at 0.11 cases per 100,000 population and account for 0.4% of all primary brain tumors (1). The most recent WHO classification of Central Nervous System (CNS) Tumors defines oligodendrogliomas as a diffusely infiltrating glioma with an *isocitrate dehydrogenase* (*IDH*) mutation and codeletion of chromosomes 1p and 19q (CNS WHO grade 2 or 3) (2).

Brain tumors have been associated with several hereditary syndromes including Lynch Syndrome (LS), which has an autosomal dominant inheritance pattern (3–5). In a study conducted to compare the incidence of brain tumors in families with LS versus that of the general population, it was found that the brain tumor incidence in LS families was 3.35% by the age of 85, compared to 0.47% in the general population (6). LS is caused by germline mutations in DNA mismatch repair (MMR) genes like *MLH1, MSH2, MSH6, PMS1*, and *PMS2*. Individuals that carry mutations in these genes have an increased susceptibility to cancers of the colon, endometrium, stomach, ovary, urinary tract, brain, skin, and many more (7–9). *PMS2* accounts for approximately 5% of the pathogenic variants involved in LS and has a lower penetrance than the other MMR genes (9–11). There is limited validated data on germline monoallelic *PMS2* mutations and, therefore, a lack of clinical and biological phenotype associations (10).

## Case presentation

**Figure 1:**
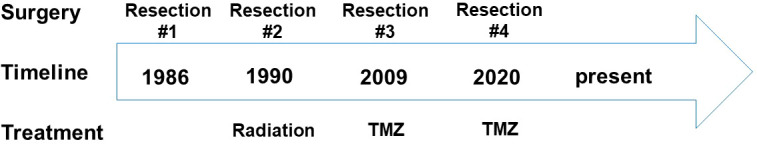
Timeline of the disease history and management. TMZ, Temozolomide.

**Figure 2:**
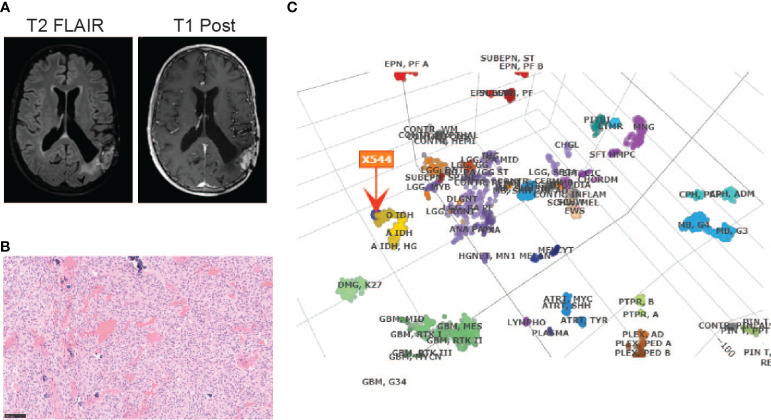
Diagnosis of an oligodendroglioma, IDH-mutant and 1p/19q co-deleted, CNS WHO grade 3 by **(A)** MRI before the most recent tumor resection; **(B)** H&E staining (20X) and **(C)** DNA methylation profiling of the tumor sample from the most recent tumor resection.

The patient is a 65-year-old gentleman diagnosed with a recurrent oligodendroglioma, IDH-mutant and 1p/19q-codeleted, CNS WHO grade 3. He was initially found to have a non-enhancing lesion in the left posterior temporal lobe at the age of 30. As illustrated in **Figure 1**, he received a craniotomy with tumor resection about thirty-six years ago. Pathological exam revealed an oligodendroglioma, grade 2. Following three years of clinical observation, he underwent a second tumor resection and received a course of radiation therapy following the surgery. His tumor remained stable until the disease progressed again twenty-three years after the initial diagnosis. At this time, the tumor was confirmed to be a recurrent oligodendroglioma, IDH-mutant and 1p/19q-codeleted, CNS WHO grade 3 after a tumor biopsy. He then received his first chemotherapy regimen, which involved 18 cycles of temozolomide. Approximately ten years later, the MRI findings were suggestive of disease progression (**Figure 2A**). He underwent a left temporoparietal craniotomy with resection of the tumor. Pathology demonstrated oligodendroglioma, IDH-mutant and 1p/19q-codeleted, CNS WHO grade 3 (**Figure 2B**). DNA methylation profiling was consistent with the diagnosis of oligodendroglioma, IDH-mutant and 1p/19q-codeleted (**Figure 2C**). He received his second regimen of chemotherapy with 6 cycles of temozolomide. The patient has been under clinical monitoring and has remained clinically stable since he completed the second round of chemotherapy.

**Figure 3:**
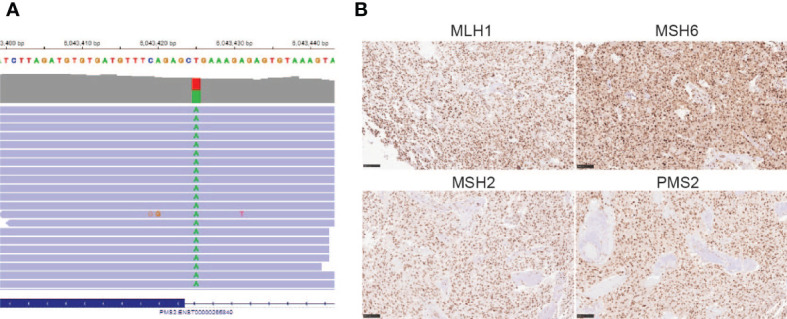
**(A)** *PMS2* mutation found in the proband (c.251-2A>T)**; (B)** Immunohistochemistry staining of MMR proteins, including MLH1, MSH6, MSH2, and PMS2 (20X) using the tumor sample from the most recent tumor resection.

**Figure 4:**
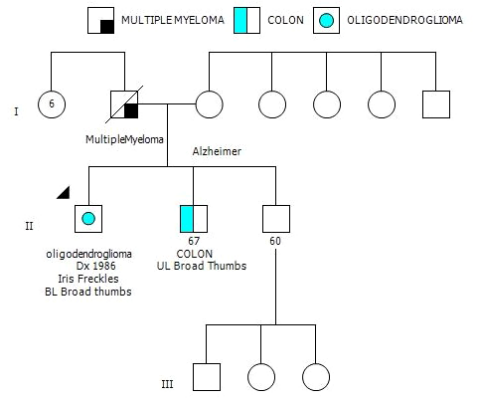
Three-generation family history pedigree.

After his most recent tumor recurrence, matched Tumor/Normal Whole Exome Sequencing (T/N WES; NCI-COMPASS-NIH) revealed that he carries a pathogenic germline mutation in *PMS2* (c.251-2A>T splice variant) (**Figure 3A**). This splice alteration affects a canonical splice acceptor at nucleotide position -2, upstream of coding exon 4 in *PMS2*. Despite the germline *PMS2* mutation, it did not cause a complete loss of PMS2 protein expression, and all MMR proteins were positively expressed (**Figure 3B**). While his family history is less characteristic of LS, he has a brother with colon cancer (**Figure 4**). The patient, however, is unable to recall further details regarding the diagnosis, including age of the diagnosis. His father also had multiple myeloma, but there are no other known cancers or hereditary syndromes in the family.

The patient was counseled on the implications of the *PMS2* germline mutation, and he was advised to undergo a colonoscopy as surveillance in accordance with the new germline finding. Although he does not have children, the patient believed that it was important for him to share the information with his brother who had a diagnosis of colon cancer. He also planned to share his genetic testing results with his primary care physician so that his medical care can be customized to this condition.

## Discussion

In this case report, we describe the case of a 65-year-old man diagnosed with a recurrent oligodendroglioma, IDH*-*mutant and 1p/19q co-deleted, CNS WHO grade 3. The tumor also harbored a pathogenic mutation in *PMS2*, a DNA MMR gene, which was determined to be a germline mutation. *PMS2* is associated with LS and in turn, with a predisposition to several types of cancers, including brain tumors.

Although uncommon, several types of brain tumors are associated with hereditary cancer syndromes like LS. Specifically, LS confers a 4-fold increased risk of developing brain tumors (12, 13). While glioblastoma is the most frequent histological subtype of brain tumors that are found in LS families (56%), other diffuse astrocytomas (22%) and oligodendrogliomas (9%) are also often found (12). Previous studies that have looked at the association between brain tumors and LS have found that 68% of patients have mutations in *MSH2*, 15% had mutations in *MLH1*, 15% in *MSH6*, and 2% in *PMS2* (12), with most showing a loss of corresponding protein expression.

Due to the reduced penetrance and genetic testing complications of monoallelic *PMS2* mutations, biallelic *PMS2* mutations are more commonly detected and better characterized, especially in constitutional MMR deficiency (CMMRD) syndrome (14–16). Several studies have examined the association between biallelic *PMS2* mutations and brain tumors (16–18). One such study reported a case of two affected sisters, one who died of a grade 3 oligodendroglioma and colorectal tumor (the proband), and the other who died of a neuroblastoma at a very early age, with no history of tumors in their parents. The proband was compound heterozygous for *PMS2* (19). Another study in a five-generation Pakistani family revealed six affected individuals who died of brain tumors at very young ages, of which three had biallelic *PMS2* mutations (20).

Carriers of monoallelic *PMS2* mutations are at an increased risk for cancer, however, less is known about monoallelic *PMS2* mutations and their association with brain tumors (21). A report focused on characterizing monoallelic *PMS2* mutations found that only 6.2% of the class 4/5 variant carriers had a family history that fulfilled the Amsterdam diagnosis criteria (10). Another study that aimed to explore the clinical phenotype of germline *PMS2* mutations uncovered that if clinicians relied solely on Amsterdam criteria to identify potential *PMS2* mutation carriers, 90.9% of the mutation carriers in their study would not have been identified, or if clinicians relied on revised Bethesda guidelines alone, 25% of the mutation carriers in their study would have been missed (22). Our patient also did not have a clear family history suggestive of LS. Despite the *PMS2* germline mutation, there was still positive protein expression in the proband, potentially describing why the patient did not have a strong LS phenotype. Moreover, retained protein expression of PMS2 also makes it hard to exclude the possibility of a coincidence in the occurrence of two moderately rare but unrelated diseases. Senter et al. further demonstrated that *PMS2*-associated LS presents highly variable clinical characteristics. For example, the age of the first LS-associated tumor in monoallelic *PMS2* carriers varies from 23-77 years (22). Since half of the carriers are diagnosed with cancer before the age of 45, the general population screening recommendations for colon cancer beginning at the age of 45 would not be adequate for families with a *PMS2* mutation.

The specific *PMS2* mutation in the patient reported here has a gnomAD allele frequency of 0.0004%, underscoring its rarity. To date, it has only been previously reported in few other cases – in an individual with suspected LS (23), in a patient with colorectal cancer and LS (10), and in a 75-year-old patient with sarcoma (24). There are multiple layers of rarity in our case – the low incidence of oligodendrogliomas diagnosed with LS, the rare association of monoallelic *PMS2* mutations with brain tumors, and the extremely low gnomAD allele frequency of the c.251-2A>T mutation in *PMS2.*

These findings emphasize that despite the uncommon nature of hereditary cancer syndromes in neuro-oncology, genetic testing and counseling can help identify genetic conditions that may require specific surveillance regimens, in turn improving patient care. Since early detection could lead to early intervention and routine surveillance, brain imaging may be indicated in some patients with LS.

## Data availability statement

The data analyzed in this study is subject to the following licenses/restrictions: a. The reported PMS2 mutation in this manuscript is from the germline whole exome sequencing. Other than this alteration, the rest of the dataset cannot be shared with the public, even by request. b. Requests to access these datasets should be directed to Jing Wu, jing.wu3@nih.gov.

## Ethics statement

The studies involving human participants were reviewed and approved by the Institutional Review Board of the National Institutes of Health. The participant provided the written informed consent to participate in this study.

## Author contributions

MM, MRay, YP, OK, ZS, and JW drafted the manuscript. MQ, MRaf, LX, JK, MT, ZA, and KA performed pathologic and molecular diagnostic testing. TP, BO, KZ, JH, TA, MG, and JW were involved in patient care. All authors contributed to the article and approved the submitted version.

## Funding

This research was supported by the NIH Intramural Research Program and Lasker Clinical Research Scholar Program. Clinical genetics and genetic counseling were supported by the NCI-CONNECT program.

## Conflict of interest

The authors declare that the research was conducted in the absence of any commercial or financial relationships that could be construed as a potential conflict of interest.

## Publisher’s note

All claims expressed in this article are solely those of the authors and do not necessarily represent those of their affiliated organizations, or those of the publisher, the editors and the reviewers. Any product that may be evaluated in this article, or claim that may be made by its manufacturer, is not guaranteed or endorsed by the publisher.
